# Gender-differences in risk factors for suicidal behaviour identified by perceived burdensomeness, thwarted belongingness and acquired capability: cross-sectional analysis from a longitudinal cohort study

**DOI:** 10.1186/2050-7283-2-20

**Published:** 2014-08-12

**Authors:** Tara Donker, Philip J Batterham, Kimberly A Van Orden, Helen Christensen

**Affiliations:** Black Dog Institute, Sydney, Australia; School of Medicine, University of New South Wales, Sydney, Australia; Department of Clinical Psychology, VU University, Amsterdam, The Netherlands; EMGO Institute for Health and Care Research, VU University and VU University Medical Center, Amsterdam, The Netherlands; Centre for Mental Health Research, Australian National University, Canberra, Australia; University of Rochester Medical Center, Rochester, USA

**Keywords:** Suicide, Gender, Risk factors, Interpersonal-Psychological Theory of Suicidal Behavior

## Abstract

**Background:**

The Interpersonal-Psychological Theory of Suicidal Behavior (IPT) is supported by recent epidemiological data. Unique risk factors for the IPT constructs have been identified in community epidemiological studies. Gender differences in these risk factors may contribute substantially to our understanding of suicidal risk, and require further investigation. The present study explores gender differences in the predictors and correlates of perceived burdensomeness, thwarted belongingness and acquired capability for suicide.

**Methods:**

Participants (547 males, 739 females) aged 32–38 from the PATH through Life study, an Australian population-based longitudinal cohort study (n=1,177) were assessed on perceived burdensomeness, thwarted belongingness and acquired capability for suicide using the Interpersonal Needs Questionnaire and Acquired Capability for Suicide Survey, and on a range of demographic, social support, psychological, mental health and physical health measures. Gender differences in the predictors of the IPT constructs were assessed using linear regression analyses.

**Results:**

Higher perceived burdensomeness increased suicide ideation in both genders, while higher thwarted belongingness increased suicide ideation only in females. In females, thwarted belongingness was uniquely related to perceived burdensomeness, while greater physical health was significantly associated with greater thwarted belongingness in males but not in females. There were trends suggesting greater effects of being single and greater perceived burdensomeness for men, and stronger effects of less positive friendship support for women associated with greater thwarted belongingness.

**Conclusions:**

Men and women differ in the pattern of psychological characteristics that predict suicide ideation, and in the factors predicting vulnerability. Suicide prevention strategies need to take account of gender differences.

## Background

One million deaths per year are estimated to occur as a result of suicide (World Health Organization (WHO) 2002). If suicide is to be prevented, we need better models of its causes and pathways. One of the leading theoretical models of suicidal behaviour is the Interpersonal-Psychological Theory of Suicidal Behavior (IPT) (Joiner 2005), which provides a testable model of suicide, and is supported by evidence from clinical, community and experimental studies (Christensen et al. 2013; Cukrowicz et al. 2011; Joiner et al. 2009; St Germain & Hooley 2013; Van Orden et al. 2008; You et al. 2011). The IPT proposes that the desire for suicide stems from two interpersonal characteristics: thwarted belongingness and perceived burdensomeness. Thwarted belongingness arises when the “need to belong” (to others) is unmet (Van Orden et al. 2010), while perceived burdensomeness refers to the belief that one is so inadequate that one`s existence is a burden on friends, family members and/or society (Van Orden et al. 2010). According to the IPT, the desire for suicide in combination with acquired capability, operationalized as a diminished fear of death and elevated physiological pain tolerance (Van Orden et al. 2010), leads to suicidal behaviour –near lethal attempts and death. Recent studies have indicated that the IPT model explained more variance in predicting suicidal behaviour than traditional mental health epidemiological models (Christensen et al. 2013); and that each of the IPT core constructs were uniquely correlated with psychological or mental health factors, such as anxiety, and psychoticism (Christensen et al. 2014). Men and women differ substantially in prevalence of suicidal behaviours. Men are more likely to die by suicide while women are more likely to attempt and survive (Bhugra 2006; Hawton & van Heeringen 2009). The pathways to suicide for males and females may be quite different, as has been recognised by many researchers (e.g., (Anestis et al. 2011; Ellis & Lamis 2007; Hawton 2000; Riley et al. 1998)). Previous research, whether directly or indirectly, has noted several gender differences in the prevalence of risk factors or correlates of suicide risk. However, gender differences in the risk factors for the IPT constructs may contribute substantially to our understanding of why particular characteristics produce increased risk of suicide for men and women and require systematic investigation. To date, the ways in which these gender differences are reflected in IPT models have not been clarified. Below we outline the evidence as to how risk factors and correlates may relate to the core IPT factors.

### Perceived burdensomeness

Poorer mental and physical health, depression, and stressful life events, such as unemployment, have been found to be associated with perceived burdensomeness (Christensen et al. 2014). Unemployment increases the risk for suicide in men but not in women (Qin et al. 2000) and may thus affect burdensomeness in males more than in females. Although mental illness increases the risk of suicide equally in both genders, higher prevalence of mental illness may confer a greater proportion of the risk for suicide in women than men (Hawton 2000; Qin et al. 2000) and thus may affect burdensomeness more in females than males. Previous research (Christensen et al. 2014) indicated that negative interactions from family and positive support from friends increased perceived burdensomeness, but this may be differentially affected by gender.

### Thwarted belongingness

Van Orden (Van Orden et al. 2010) has suggested that women may be particularly sensitive to thwarted belongingness because of the importance of close ties and family for happiness. The significant higher rates of suicide attempts among female immigrants in Europe and other Western countries compared to non-Western countries, especially in young women moving from traditional to more individualistic societies suggests higher thwarted belongingness (Van Bergen et al. 2008; Burger et al. 2002; Bursztein Lipsicas et al. 2013; Miranda et al. 2013). Marriage in itself appears to be a protective factor for men, whereas in married women, being a parent of a young child was found to be a protective factor for suicide risk (Hawton 2000; Qin et al. 2000). For unmarried men, thwarted belongingness may therefore be higher.

### Acquired capability

Several studies have indicated that men possess significantly higher levels of pain tolerance (Riley et al. 1998) and lower levels of fear of suicide (Ellis & Lamis 2007), which are measures of the two facets of acquired capability. Previous findings also indicate a direct relationship between male gender and increased acquired capability (Van Orden et al. 2008; Christensen et al. 2999; Anestis et al. 2011). In addition, Anestis et al. (Anestis et al. 2011) found that distress tolerance interacted with sex to predict acquired capability, such that males with high distress tolerance had the highest levels of acquired capability.

The present study explores whether there are gender differences in the predictions of the IPT model with respect to both suicidal ideation and suicidal behaviours. The aims of the present study are twofold. First, to examine the IPT model separately for men and women, to determine if the same relationship holds in men and women. Secondly, to assess predictors of these core constructs separately for men and women, to determine whether the same risk factors predict higher scores on each of the core IPT constructs. The risk factors examined were gender, age, years of education completed, marital status, number of recent stressful events, number of lifetime traumas, positive and negative interactions from friends and family, ruminative style, mastery, personality traits, physical and mental health scores, self-reported presence of generalized anxiety and presence of major depression. The risk factors included either have direct associations with the IPT constructs, or have associations with suicide ideation (e.g. rumination (Vilhjalmsson et al. 1998), Mastery (Blüml et al. 2013), personality traits (Martin et al. 2004) and life-time traumas (Merrill & Owens 1986).

## Methods

### Participants and procedure

The PATH Through Life Project is a population-based study examining the health and well- being of people who were initially 20–24, 40–44, and 60–64 years of age (Anstey et al. 2012). Each cohort is being followed up every four years over a total period of 20 years. Participants were randomly sampled from the electoral rolls for the city of Canberra, Australia, and in the neighbouring town of Queanbeyan. Results presented here concern only the 20s cohort, with data from the fourth wave of interviews conducted in 2011–2012, when participants were aged 32–38 (the majority being 33–37). The rationale for including only this cohort cross-sectionally is because the INQ items were only included for that cohort and only at the most recent assessment. At the first wave, interviews were completed with 2,404 in the 20–24 year age-group, of which, 1242 (51.7%) were female and 1162 (48.3%) were male. The participation rate of those who were found to be in the appropriate age range was 58.6%. Follow-up interviews were completed by 1,191 (49.5%) participants (42.5% male, 57.5% female) at wave 4, 12 years after the initial interview, with a further 95 partial completions (53.5% total). The response rate was markedly lower than for previous interview waves (88.6% and 79.7% at Waves 2 and 3), due to reduced project funding that required the interviews be largely conducted online. At Wave 3, there were no significant differences in the rates of suicidal ideation ( = 1.65, p = 0.199), suicidal behaviours ( = 0.02, p = 0.881), presence of anxiety ( = 3.41, p = 0.065) or presence of depression ( = 1.56, p = 0.212) between those who did and did not complete Wave 4. Those who completed Wave 3 but not Wave 4 had significantly less education (14.0 vs 14.4 y; F _1, 1964_ = 35.1, p < 0.001) and females had significantly higher rates of assessment completion (68% of F vs 58% of M;  = 22.2, p < 0.001). After exclusion on the basis of missing outcome data was made, the sample size for the regression analyses was n=1,177. Items used in the present analyses were based on a self-completed online survey. Approval for the research was obtained from The Australian National University’s Human Research Ethics Committee (protocol #2010/542). All participants provided written informed consent to participate in the study.

### Measures

The suicidal ideation outcome was based on endorsement on one yes/no item from the Psychiatric Symptom Frequency scale (Lindelow et al. 1997): “In the last year have you ever thought about taking your own life?”. The IPT constructs of perceived burdensomeness and thwarted belongingness were assessed using seven and five items respectively from the Interpersonal Needs Questionnaire (INQ) (Van Orden et al. 2008). The INQ, derived from the Interpersonal Theory of Suicide, was developed to measure thwarted belongingness and perceived burdensomeness. A validation study by Van Orden et al. (Van Orden et al. 2012) supported the two constructs being distinct but related and reliable. An example item of the INQ is “These days the people in my life would be better off if I were gone”. These items are rated on a seven-point scale from 1 “Not at all true for me”, through 4 “Somewhat true for me”, to 7 “Very true for me”, with scores based on the mean item response ranging from 1–7. Acquired capability for suicide was assessed using five items of the Acquired Capability for Suicide Scale (ACSS) (Van Orden et al. 2008). An example item of the ACSS is: “Things that scare most people don't scare me”. Responses for these items are rated from 0 “Not at all like me” to 4 “Very much like me”, with the acquired capability score assessed as the mean of items, ranging from 0–4. The scale showed good reliability, discriminant and convergent validity (Van Orden et al. 2008). The risk factors examined were gender, age, years of education completed, marital status, number of recent stressful events, number of lifetime traumas, positive and negative interactions from friends and family (Schuster Social Support Scale; (Schuster et al. 1990)), ruminative style (Butler & Nolen-Hoeksema 1994), mastery (Pearlin & Schooler 1978), personality traits, SF-12 physical and mental health scores (Ware et al. 1996), self-reported presence of generalized anxiety and presence of major depression. All questionnaires showed acceptable to good psychometric properties (Schuster et al. 1990; Butler & Nolen-Hoeksema 1994; Pearlin & Schooler 1978; Ware et al. 1996; Rosenman 2002). A count of stressful events in the past six months was identified from a list of 16 events: suffered illness/injury/assault, relative suffered illness/injury/assault, parent/child/partner died, close family friend/relative died, broke off a relationship, serious problem with friend/neighbour/relative, career crisis, thought would soon lose job, partner thought they would soon lose job, partner had career crisis, marriage separation, unemployment, being fired, financial crisis, legal problems, or having something valuable lost or stolen. Lifetime traumas were assessed as a count of adverse experiences from 10 items, including combat experience, life- threatening accident, natural disaster, witnessing injury or death, rape, sexual molestation, physical attack or assault, being threatened with a weapon/held captive/kidnapped, being tortured or a victim of terrorism, or other extremely stressful/upsetting event (Rosenman 2002). Social support was assessed using summed measures of both negative and positive support from family and friends (Schuster et al. 1990). The items were “How often do friends make you feel cared for?”, “How often do friends express interest in how you are doing?”, “How often do family make you feel cared of?” and “How often do family express interest in how you are doing?” Responses were given on a four-point scale ranging from “often” to “never”. Three personality traits of neuroticism, extroversion and psychoticism were measured at the initial interview, twelve years before the outcome data were assessed, using the Eysenck Personality Questionnaire-Revised (Eysenck et al. 1985). These traits tend to be highly stable (four-year reliability correlations of 0.56-0.74). Presence of major depressive episode was assessed using the nine-item Patient Health Questionnaire (PHQ-9(Spitzer et al. 1999)) based on the algorithm identified by the authors of the scales, specifically, presence of anhedonia or feelings of depression (first two items of PHQ-9) and five or more of the nine PHQ-9 items being rated as “more than half the days” or higher (or “several days” or higher for the suicidal ideation item). An example item is “How often have you been bothered by little interest of pleasure in doing things?”. In a review of Wittkampf et al. (Wittkampf et al. 2007), a sensitivity of 0.77 (0.71–0.84) and a specificity of 0.94 (0.90–0.97) was found for the PHQ-9. Presence of Generalized Anxiety Disorder was assessed using the GAD-7 scale (Spitzer et al. 2006), which was also scored using the authors’ diagnostic algorithm (see (Spitzer et al. 1999)) based on ratings of “more than half the days” or “nearly every day” on the first item and at least three subsequent items. An example item is “how often have you been bothered by feeling nervous, anxious or on edge?”. Reliability and validity are excellent (Cronbach's α=0.92, AUC: 0.91). With a cut-off point of ≥10, sensitivity is 0.89 and specificity is 0.82 among primary care participants (Eysenck et al. 1985). Both the PHQ-9 and the GAD-7 are based on past two weeks.

### Analysis

Two exploratory factor analyses were conducted, the first with items from the shortened version of the INQ, and the second with the shortened items of the ACSS. Descriptive statistics for the sample were tabulated by gender. Differences in potential risk factors between female and male participants with and without suicidal behaviour were assessed using F values from one- way ANOVAs and chi-square statistics for continuous and categorical variables respectively. Next, logistic regression analyses were used to test the predictions of the IPT model for suicidal ideation in males and females. The variables were centred to reduce multicollinearity. The independent variables for the model were the risk factors hypothesized by the IPT to predict ideation (perceived burdensomeness, thwarted belongingness and the interaction of these two constructs). The rates of suicidal ideation based on levels of perceived burdensomeness and thwarted belongingness were plotted using tertile splits of scores on these constructs. The logistic regression model for plans and attempts were not tested because prevalence was too low (n= 18, 1.4%). The hypothesized interaction between acquired capability and ideation could not be tested in the model for plan/attempts, as all participants who reported a plan or attempt also experienced ideation. As thwarted belongingness and perceived burdensomeness are distinct, but related constructs (Martin et al. 2004), we included the constructs as covariates in each of the models to assess risk factors for each of the constructs independent of the other constructs. Linear regression models were used to assess the association between the potential risk factors and the three IPT constructs: perceived burdensomeness, thwarted belongingness and acquired capability for suicide in separate models for males and females. We tested whether the models for males explained more variance than the models for females using Chow tests (Chow 1960). To test the accuracy of the model for males compared to females, area under the ROC curve analysis (Hanley & McNeil 1982) were conducted. Finally, where possible differences were observed in the effects for males and females (i.e., one significant and the other non-significant, or both significant but of different magnitude), we examined a linear regression model that included the effects of gender, the risk factor and the interaction between gender and the risk factor. We then interpreted discrepant effects as indicating gender differences only when this interaction term was significant.

SPSS version 20 was used for all analyses. Because of the exploratory nature of the analyses, alpha was set at P<.01.

## Results

### Construct validity of the INQ and ACSS

A single factor analysis of the INQ found that the seven burden items loaded on a single factor (based on scree plot) explaining 59.7% of variance, with factor loadings of >0.75 with the exception of item 5 (loading 0.38). A separate factor analysis of the ACSS found that the five belonging items loaded on a single factor explaining 60.0% of the variance, with each item loading >0.64. The five ACSS items loaded on a single factor accounting for 44.5% of variance, with loadings >0.78 for the pain tolerance items and >0.35 for the fear of death items.

### Gender differences in the IPT risk factors

Only results of gender differences at P<.01 are reported. The community cohort of 32–38 year olds had a mean age of 34.7 and 58% of respondents were female. Sample characteristics are presented in Table 1 by gender. There were no gender differences in perceived burdensomeness or thwarted belongingness, but males reported significantly higher levels of acquired capability for suicide compared to females. Furthermore, compared to males, females experienced significantly higher levels of positive support from friends, higher levels of negative interactions from family, higher ruminative style, neuroticism and PHQ-9 depression. Males expressed higher levels of mastery, psychoticism, SF-12 physical and mental health. There were no differences in suicidal ideation or plans/attempts between males and females.

**Table 1:** **Descriptive statistics by gender**

	Males (n=547)		Females (n=739)			
	Mean	SD	Mean	SD	F	P
Perceived burdensomeness	1.47	0.72	1.55	0.86	2.70	.101
Thwarted belongingness	2.24	1.29	2.10	1.23	3.79	.052
Acquired capability for suicide	2.80	0.80	2.46	0.84	52.18	**<.001**
Age	34.69	1.47	34.65	1.52	0.19	.666
Years of education	14.31	1.38	14.52	1.52	6.50	.011
Number of stressful events	1.13	1.48	1.21	1.43	1.01	.316
Number of lifetime traumas	1.40	1.50	1.26	1.38	3.00	.084
Positive support from friends	4.74	1.24	5.15	1.16	35.85	**<.001**
Negative interactions from friends	2.88	1.69	2.76	1.62	1.67	.196
Positive support from family	5.37	1.11	5.32	1.18	0.54	.461
Negative interactions from family	3.61	1.99	4.11	2.16	17.16	**<.001**
Ruminative style score	7.89	5.93	10.20	6.27	41.91	**<.001**
Mastery score	22.35	3.54	21.78	3.50	7.60	.006
Neuroticism (wave 1)	3.79	3.17	5.46	3.35	81.37	**<.001**
Extroversion (wave 1)	7.99	3.47	8.29	3.44	2.31	.129
Psychoticism (wave 1)	2.94	1.76	2.34	1.69	36.95	**<.001**
SF-12 physical health	52.92	6.51	51.18	7.82	17.66	**<.001**
SF-12 mental health	49.09	9.52	47.26	9.95	10.87	**<.001**
	Frequency	Percent	Frequency	Percent	χ^2^	p
Presence of suicidal ideation	47	8.9%	64	8.9%	0.00	.965
Presence of plan/attempt	6	1.1%	12	1.6%	0.63	.426
PHQ-9 clinically significant major depression	23	4.4%	69	9.6%	12.10	**<.001**
GAD-7 clinically significant generalized anxiety disorder	20	3.8%	39	5.4%	1.78	.182
Marital status					5.86	.053
Married	335	61.4%	415	56.2%		
Separated/divorced/widowed	26	4.8%	56	7.6%		
Single, never married	185	33.9%	268	36.3%		

*Note:* **bold** values indicate P < 0.01; SF-12: Short Form-12; PHQ: Patient Health Questionnaire; GAD: Generalized Anxiety Disorder.

**Figure 1:**
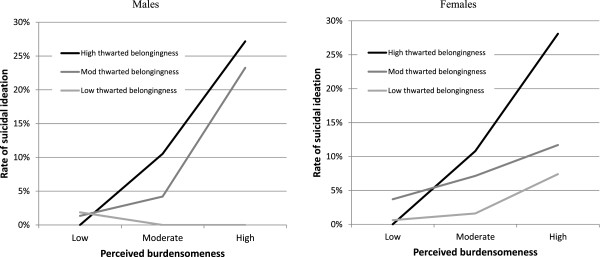
**Rates of suicidal ideation by gender, based on tertile splits of thwarted belongingness and perceived burdensomeness.**

Table 2 and Figure 1 show data examining gender differences in the IPT. Each point of increase in perceived burdensomeness on the seven-point scale was associated with approximately a five-fold increase in the odds of experiencing thoughts of suicide for males, and a two-fold increase for females. A one-point increase in thwarted belongingness increased the odds of suicide ideation by 67% in females, while for males this increase was not significant. Based on area under the ROC curve, the model was not significantly more accurate for males compared to females (AUC male = 0.827, AUC female = 0.825, P= .97 (Spitzer et al. 1999)). Based on separate linear regression analysis, The variance explained by the perceived burdensomeness model were similar for males and females (males: Adjusted *R*^*2*^= 0.53; females: adjusted *R*^*2*^= 0.53; *F*(24, 1118) = 0.81, P = .73). However, more variance was explained in the thwarted belongingness model for females and less variance was explained in the thwarted belongingness model for males (males: Adjusted *R*^*2*^= 0.53; females: adjusted *R*^*2*^= 0.58; *F*(24, 1118) = 1.95, P = .004). More variance was explained in the acquired capability model for males, while less variance was explained in the acquired capability model for females (males: Adjusted *R*^*2*^= 0.17; females: adjusted *R*^*2*^= 0.12; *F*(24, 1118) = 2.12, P = .001).

**Table 2:** **Logistic regression models of suicidal ideation based on the Interpersonal-Psychological Theory of suicidal behaviour**

	Males (n = 520)		Females (n = 713)	
	OR	P	OR	P
Perceived burdensomeness	4.874	**<.001**	2.115	**<.001**
Thwarted belongingness	1.227	.301	1.674	**<.001**
Burden x belonging	0.731	.019	0.893	.212
Constant	0.063	<.001	0.064	<.001

The results from multivariate linear regressions in gender differences for each of the three core constructs (perceived burdensomeness, thwarted belongingness and acquired capability for enacting suicide) are presented in Table 3.

**Table 3:** **Linear regression models examining gender differences in the predictors of the three Interpersonal-Psychological Theory constructs (n male = 491,n** ***female = 676)***

	Perceived burdensomeness				Thwarted belongingness				Acquired capability			
	Males		Females		Males		Females		Males		Females	
	Estimate	P	Estimate	P	Estimate	P	Estimate	P	Estimate	P	Estimate	P
(Constant)	2.304	.001	1.777	.009	3.430	.006	5.335	<.001	2.753	.008	0.084	.927
Age	0.000	.999	0.013	.396	0.008	.772	0.002	0.921	−0.022	.359	0.001	.961
Years of education (wave 1)	−0.019	.288	−0.004	.798	−0.034	.283	−0.020	0.360	0.018	.506	0.033	.122
Marital status												
Married (reference)	--	--	--	--	--	--	--	--	--	--	--	--
Separated/divorced/widowed	−0.115	.306	0.117	.186	0.315	.110	−0.115	.332	−0.055	.740	0.138	.243
Single, never married	−0.042	.425	0.026	.604	0.262	**.004**	0.146	.028	−0.032	.676	0.186	.005
Burdensomeness					0.570	**<.001**	0.435	**<.001**	0.059	.389	0.064	.224
Belongingness	0.185	**<.001**	0.242	**<.001**					−0.021	.590	0.029	.454
Acquired capability	0.027	.389	0.036	.224	−0.030	.590	0.029	.454				
Number of life events	0.040	.020	−0.006	.738	−0.010	.744	−0.009	.714	0.053	.035	0.045	.064
Number of life traumas	−0.002	.916	−0.001	.974	−0.021	.463	0.009	.726	0.093	**<.001**	0.108	**<.001**
Positive support from friends	0.056	.014	0.011	.668	−0.266	**<.001**	−0.313	**<.001**	−0.010	.764	0.019	.576
Negative interactions from friends	−0.011	.479	−0.035	.029	0.083	.003	0.040	.064	0.051	.030	0.053	.013
Positive support from family	−0.041	.091	−0.014	.559	−0.222	**<.001**	−0.148	**<.001**	−0.114	**<.001**	0.002	.950
Negative interactions from family	0.011	.435	0.030	.019	0.072	.003	−0.030	.077	−0.043	.036	0.007	.688
Ruminative style	0.013	.022	0.008	.123	0.001	.931	0.012	.077	0.002	.771	−0.005	.433
Mastery	−0.020	.012	−0.016	.053	−0.019	.180	−0.045	**<.001**	0.031	**.009**	0.024	.029
EPQ neuroticism (wave 1)	0.002	.831	0.009	.272	0.011	.485	0.016	.154	−0.034	**.009**	−0.027	.017
EPQ extroversion (wave 1)	−0.014	.062	−0.001	.914	−0.002	.887	−0.002	.826	0.019	.098	0.005	.627
EPQ psychoticism (wave 1)	0.005	.724	−0.002	.910	0.038	.107	0.026	.161	0.088	<.001	0.048	.010
SF12 physical	−0.003	.464	−0.007	.029	0.016	.017	−0.001	.869	−0.006	.296	0.003	.471
SF12 mental	−0.012	**.00**	−0.015	**<.001**	−0.009	.170	−0.014	**.003**	0.005	.331	0.010	.031
PHQ generalised anxiety	−0.382	**.006**	−0.234	.017	0.264	.285	0.101	.440	0.194	.347	−0.145	.267
PHQ major depression	1.095	**<.001**	1.121	**<.001**	0.003	.991	0.105	.554	0.250	.291	0.008	.966
Past ideation (waves 1-3)	0.103	.139	0.043	.509	0.065	.597	0.017	.850	0.242	.018	0.154	.080
Past attempt (waves 1-3)	0.283	.193	0.234	.133	−0.233	.542	−0.192	.358	0.240	.451	0.044	.831

*Note:* **bold** values indicate P < .01; SF-12: Short Form-12; PHQ: Patient Health Questionnaire; GAD: Generalized Anxiety Disorder.

#### Perceived burdensomeness

In the multivariate linear regression, the absence of Generalised Anxiety (GAD) predicted higher perceived burdensomeness only in males, although follow-up analyses found no significant interaction effect between GAD and gender on perceived burdensomeness. However, a significant gender interaction effect was found for the effect of thwarted belongingness (t = 3.70, P < 0.001) indicating a greater effect of thwarted belongingness on perceived burdensomeness for females than males. The model in Table 3 also shows significant effects of SF-12 mental health and PHQ-9 depression, although these were not significantly different by gender.

#### Thwarted belongingness

Males but not females experiencing higher levels of negative interactions from friends and family or being single/never married expressed higher levels of thwarted belongingness, whereas lower mastery levels and poorer mental health was associated with increased thwarted belongingness in females but not males. However, follow-up linear regression models testing the interaction between gender and each risk factor separately found that none of these effects were significant at P< 0.01, although there were trends suggesting greater effects of being single (t = −2.27, P= 0.023) and greater perceived burdensomeness (t = −2.39, P= 0.017) for men, and stronger effects of less positive friendship support for women (t = −2.33, P= 0.023). In addition, although SF-12 physical health was not significant at P < 0.01 for males or females, there was a significant gender interaction indicative of stronger effects of greater physical health on greater thwarted belongingness among men than women (t = −3.41, P= 0.001).

#### Acquired capability

Less positive support from family, higher levels of mastery and psychoticism and lower levels of neuroticism were associated with higher levels of acquired capability in males but not in females. Only among females, significantly higher levels of acquired capability were seen among those who were single or never married. However, follow-up linear regression models testing the interaction between gender and each risk factor separately found that none of these interactions were significant. Life traumas were significantly associated with greater acquired capability for both men and women.

## Discussion

The present study is, as far as we know, the first study testing the predictions of the IPT model for gender differences in a community-based cohort. Furthermore, no other study has directly identified gender differences in the predictors associated with the core IPT constructs of thwarted belongingness, perceived burdensomeness and acquired capability for suicide.

### Partial support for the IPT model

There was partial support for the IPT model. Higher levels of perceived burdensomeness was associated with increased suicidal ideation in both genders, whereas thwarted belongingness was associated with increased suicidal ideation in women only. High levels of perceived burdensomeness increased risk for suicidal ideation, particularly among males.

There was no gender difference in the variance explained by the perceived burdensomeness model. More variance was explained for females than males for thwarted belongingness while more variance was explained for males than females for the acquired capability model. However, especially for acquired capability, relatively little variance was explained by the model.

### Gender differences in predictors

Because of the exploratory nature of the analyses, we only discuss results of p-values of <.01 and results from follow-up analyses.

### Perceived burdensomeness

Previous research found strong evidence for the interaction of thwarted belongingness and perceived burdensomeness increasing the risk of suicide ideation (Christensen et al. 2013; Joiner et al. 2009; Van Orden et al. 2008). Results from our study indicated a greater effect of thwarted belongingness on perceived burdensomeness for females but not males. This is an important finding as women who experience feelings that they do not belong may be at higher risk for experiencing increased feelings of burdensomeness, leading to greater risk for suicide ideation. Previous research has not examined potential pathways whereby the theory`s interpersonal constructs may influence each other; this is a novel contribution of the current study.

### Thwarted belongingness

The present study showed a trend that in single or unmarried males but not females, thwarted belongingness was higher. This is in line with previous research (Hawton 2000; Qin et al. 2000), indicating that marriage in itself appears to be a protective factor for men. This study also found a trend that a higher level of perceived burdensomeness affected thwarted belongingness more in males than females.

Our results suggest that the etiological pathways in the IPT are likely more complex than originally proposed. In particular, the effects of belongingness and burdensomeness do not appear to be independent, and depend on gender. For women, the model could include a causal link from belongingness to burdensomeness and for men, the model could include a causal link from burdensomeness to belongingness. A frequently raised question regarding the theory is whether an individual can experience a feeling of disconnection (e.g., thwarted belonging and at the same time experience connectedness- albeit negative in the form of burdensomeness). Our results are consistent with the theory`s proposal that one can experience both constructs simultaneously, while also generating a hypothesis that these constructs fuel each other, although in different directions for men and women. Future research examining the pathways among the construct of the IPT is needed, especially with regards to gender. For example, the theory is silent as to whether belongingness and burdensomeness might influence acquired capability. However, given research indicating that social pain activates the same neural pathway as physical pain, it may very well be the case that all of the IPT construct are interrelated.

Furthermore, less positive friendship support affected thwarted belongingness more in females than males, and thus may confer a greater proportion of the risk for suicide in women than men. Interestingly, a greater physical health affected thwarted belongingness more among men than women. An interaction effect with depression might be possible, but future research will be needed to test this.

### Acquired capability

In the present study, no interaction effects of gender on acquired capability were found.

### Limitations

The strengths of this study include a large community-based cohort and the inclusion of a large set of potential risk factors. However, this study also had several limitations. First, because of the independent samples, we were not able to test whether the models of perceived burdensomeness, thwarted belongingness and acquired capability significantly differed from each other for males or females. There also may have been subgroup differences within each gender. Second, due to low suicide ideation and plans/attempts base rates of the sample, there may have been insufficient power to detect true gender differences in the IPT constructs. However, males had higher levels of acquired capability, consistent with previous research (Anestis et al. 2011). Third, we were not able to include risk factors which have previously been identified to differentiate between gender effects, such as distress tolerance (Anestis et al. 2011) or migration (Bursztein Lipsicas et al. 2013), and other low prevalence risk factors, such as sexual abuse (Martin et al. 2004). Fourth, although some risk factors were measured longitudinally, the analysis used a cross-sectional design. Therefore it was not possible to determine whether most risk factors were the cause rather than the consequence of suicidal behaviour. Further research using a longitudinal design (such as research on the present cohort after the next follow-up in 2015) would provide stronger research evidence. Fifth, due to power limitations and difficulties of interpreting multiple interactions, we did not include all covariates in the models to test the interaction of gender with each risk factor. Finally, we did not have access to data on completed suicides, and there are likely to be very few suicides even in a cohort of this size. Therefore, our conclusions on acquired capability may be limited to individuals in the community with capability to engage in non-lethal suicidal behaviour.

### Clinical implications

Our findings suggest that risk factors for perceived burdensomeness and thwarted belongingness differ across genders. In females, thwarted belongingness was uniquely related to perceived burdensomeness, while greater physical health was significantly associated with greater thwarted belongingness in males but not females. This study contributes to a further understanding of the complex psychological and societal differences in women and men affecting the three IPT constructs for suicidal behaviour. Identifying gender differences may enhance predictive value of risk factors for the IPT constructs of suicidal behaviour, and accordingly, allow for more precise identification of those being at risk of suicide. In addition, developing different suicide prevention strategies or interventions according to gender differences may enhance effectiveness and dissemination of those treatments. This study found that females may benefit more when screening and treatment is focused at increasing belongingness through targeting its associated risk factors (e.g., support from friends). Suicide prevention strategies focusing on decreased burdensomeness through targeting relationship status may reduce suicidal ideation for males in particular. Nevertheless, burdensomeness had a large effect on suicidal ideation in both males and females, which indicates that non-gender-specific risk factors play an important role in precipitating suicidality. However, given the explanatory natures of our cross-sectional analysis, results need to be replicated in future research.

### Further research

It is very likely that the risk factors associated with the three IPT constructs have complex interactions with many other clinical and sociodemographic variables. Examination of these possible interactions is a challenge for future research. In addition, a very important area for further research involves the need to detect which of the numerous strategies to decrease levels of perceived burdensomeness, thwarted belongingness and acquired capability are most effective for males and females to meet their individual needs.

## Conclusions

In sum, men and women may differ in the pattern of psychological characteristics that predict suicide ideation, and in the factors that predict vulnerability. Suicide prevention strategies need to take account of gender differences. For females, suicide prevention strategies that aim to increase support from friends may be more effective. Interventions aiming to reduce suicidal ideation in males may be more effective when they focus on building skills to feel less burdened.

## Abbreviations

ACSS: Acquired capability for suicide scale
ANOVA: Analysis of variance
AUC: Area under the ROC curve
GAD: Generalized anxiety
GAD-7: Generalized anxiety disorder
INQ: Interpersonal needs questionnaire
IPT: Interpersonal-psychological theory of suicidal behavior
PHQ-9: Patient health questionnaire
ROC: Receiver operating characteristics
SF-12: Short-form health survey −12
SPSS: Statistical package for the social sciences.
